# Protein pyrophosphorylation: moving forward

**DOI:** 10.1042/BCJ20160710C

**Published:** 2016-10-27

**Authors:** Adolfo Saiardi

**Affiliations:** Medical Research Council Laboratory for Molecular Cell Biology, University College London, Gower Street, London WC1E 6BT, U.K.

## Abstract

Genetic ablation of inositol pyrophosphate synthesis has established the fundamental importance of this class of molecules to the eukaryote cell. These studies, however, must be complemented by cell biology and biochemical approaches to appreciate the signalling involved in the processes regulated by inositol pyrophosphates. A recent study by Chanduri et al. published in the *Biochemical Journal*, by integrating multiple experimental approaches, demonstrated that inositol pyrophosphates regulate intracellular vesicular movement. In particular, the vesicular transport along the microtubule that is driven by the motor protein complex dynein. Importantly, one subunit of this cellular motor, dynein 1 intermediate chain 2, undergoes serine pyrophosphorylation, a post-translational modification driven by inositol pyrophosphates. The pyrophosphorylation status of this dynein intermediate chain regulates its interaction with dynactin, which recruits the motor to vesicles. This mechanistically might explain how inositol pyrophosphates control intracellular membrane trafficking. By dissecting the serine pyrophosphorylation process, this work increases our awareness of this modification, underappreciated by the scientific literature but probably not by the eukaryotic cell.

The ever-growing interest in the inositol pyrophosphates as signalling and metabolic messengers is fully justified by the many unique and exciting features this class of molecule possesses. Inositol pyrophosphates are ubiquitously present in eukaryotes, turn over very rapidly and, most importantly, contain one or more high-energetic pyrophosphate moiety/ies, hence their name [1]. Furthermore, this class of molecules appears to regulate a wide range of cell biological processes, and thus it has been proposed that inositol pyrophosphates might control a fundamental cellular function. Studies indicate that inositol pyrophosphates regulate basic metabolism [1,2] such as cellular energetics [3] or phosphate homeostasis [4,5]. While there is a continued proliferation of processes known to be regulated by inositol pyrophosphates, the most intriguing and controversial aspect of this research field is the mechanism/s of action by which this class of molecules exercise its functions [6]. Inositol pyrophosphate may affect protein function allosterically by binding; this represents the accepted mechanism of action of other inositol polyphosphates such as inositol trisphosphate (IP_3_), which releases calcium from intracellular stores by binding to the IP_3_ receptor. However, while there is a growing number of proteins shown to have inositol pyrophosphate-binding capability, the specific and selectivity of this process is vulnerable given the extraordinary charge density possessed by this class of molecules. An alternative mechanism of action relies on the distinctive presence of the pyrophosphate moiety. In this model (Figure 1), an ATP kinase, usually casein kinase 2 (CK2), pre-phosphorylates and primes a target serine residue. Subsequently, the hydrolysis of the highly energetic pyrophosphate moiety drives the non-enzymatic transfer of the β-phosphate to the phosphorylated serine, forming a pyrophosphoserine [7,8]. The two mechanisms of action are not mutually exclusive and can coexist in cells. Studying protein pyrophosphorylation is technically challenging. First and foremost, it requires the synthesis of ^32^P-radiolabelled inositol pyrophosphates such as radiolabelled 5-diphosphoinositol pentakisphosphate (5-[β-^32^P]IP_7_); although straightforward, preparing one's own radiolabelled chemicals requires a commitment not commonly found in today's young researchers. Consequently, any effort aiming to study protein pyrophosphorylation is commendable, including the report by Chanduri et al. [9] published in a recent issue of the *Biochemical Journal*.

**Figure 1.**
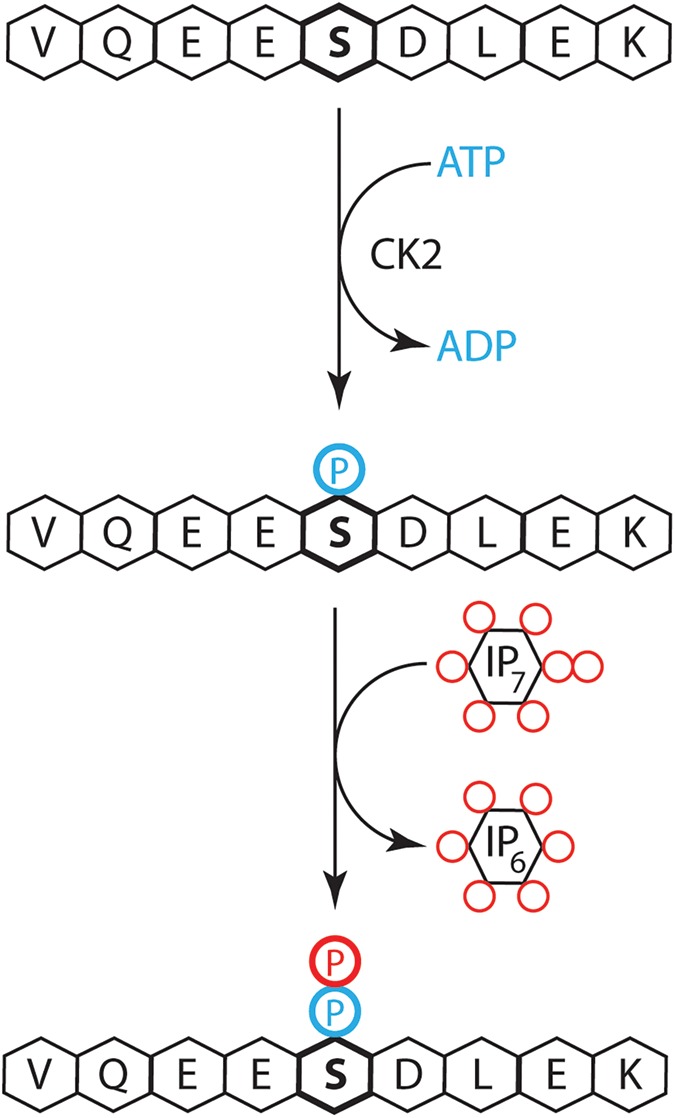
Protein pyrophosphorylation is generate by a two-step mechanism. The serine residue (S, bold) is initially phosphorylated by CK2 that generates a phosphoserine residue using ATP (P, blue). Subsequently, inositol pyrophosphates, such as diphosphoinositol pentakisphosphate (IP_7_) (P, red), transfer the β-phosphate of the pyrophosphate moiety to the phosphoserine in a non-enzymatic manner, generating a pyrophosphoserine residue and inositol hexakisphosphate (IP_6_).

Chanduri et al. [9] mainly, but not exclusively, used the genetic model of mouse embryonic fibroblasts (MEFs) derived from *ip6k1^−/−^* mice in their study. The knockout of IP6K1 (inositol hexakisphosphate kinase 1) results in a decrease of inositol pyrophosphate levels in *ip6k1^−^*^/*−*^ MEFs [10]. Using a classical experimental approach, namely transferrin uptake, it was observed that while transferrin's immediate entry was unaffected, its accumulation in the perinuclear endocytic recycling compartment (ERC) was dramatically reduced in *ip6k1^−/−^* MEFs. Studying the co-localization of transferrin with the early endosomal marker EEA1 revealed that the degree of EEA1–transferrin co-localization was higher in *ip6k1^−^*^/*−*^ MEFs compared with *ip6k1^+/+^* MEFs (wild-type, WT). This indicates that transferrin's exit from early endosomes is delayed in *ip6k1^−^*^/*−*^ MEFs. The expression of active, but not inactive, IP6K1 rescued these defects, demonstrating that inositol pyrophosphates are required for endosomal sorting of transferrin and its accumulation in the ERC. Furthermore, Chanduri et al. [9] have tracked vesicular movement *in vivo* using a specific fluorescence sensor, demonstrating that the speed of fluorescent endosomes is significantly lower in *ip6k1^−^*^/*−*^ MEFs. Another assay used by the authors to demonstrate altered intracellular vesicular movement is phagosome distribution. After allowing latex beads to be phagocytized by primary macrophages isolated from *ip6k1^−^*^/*−*^ and WT mice, a greater number of phagosomes in nuclear proximity was observed in macrophages collected from WT mice [9].

Phagosome movement and transferrin transport from early endosomes to the ERC occur along cytoskeletal microtubules in a dynein-dependent manner. The motor protein dynein comprises two heavy chains with ATPase activity, responsible for generating the movement, and several smaller proteins important in stabilizing the macromolecular complex, and for cargo docking. One of these proteins, cytoplasmic dynein 1 intermediate chain 2 (DYNC1I2 or IC-2C), possesses a pyrophosphorylation consensus sequence characterized by one or more serines embedded in a streak of acidic amino acids [7]. This type of sequence also represents the canonical phosphorylation site for CK2, an event necessary to pre-phosphorylate and prime the target serine for pyrophosphorylation [7]. Mutagenesis of this putative target site (serine-51 in mouse IC-2C sequence) to alanine abolished IC-2C pyrophosphorylation. However, the mutagenesis also abolished the CK2 pre-phosphorylation event. Therefore, to overcome this problem and to assess whether IC-2C undergoes pyrophosphorylation *in vivo*, the authors carried out a ‘back-pyrophosphorylation’ assay [11]. The logic of this assay relies on the assumption that pyrophosphorylation target proteins isolated from WT MEFs are already pyrophosphorylated *in vivo*, whereas target proteins isolated from *ip6k1^−^*^/*−*^ MEFs are not. Consequently, on performing the back-pyrophosphorylation assay *in vitro* on proteins purified from WT or *ip6k1^−^*^/*−*^ MEFs, these target proteins will show a differential incorporation of radiolabelled phosphate inversely proportional to the amount of *in vivo* pyrophosphorylation. Indeed, incubation of radiolabelled inositol pyrophosphate 5-[β-^32^P]IP_7_ with immunoprecipitated IC-2C resulted in virtually no pyrophosphorylation of WT-derived IC-2C, indicating that the protein is *in vivo* pyrophosphorylated and cannot be further pyrophosphorylated *in vitro*. Conversely, IC-2C immunoprecipitated from *ip6k1^−^*^/*−*^ MEF extracts was promptly radiolabelled *in vitro*, indicating that in these cells this protein is poorly pyrophosphorylated or non-pyrophosphorylated [9].

How does IC-2C pyrophosphorylation status affect vesicular movement? The IC-2C pyrophosphorylation site is located in a region that interacts with the subunit p150^Glued^ of the dynactin complex. The authors demonstrated using pull-down experiments as well as co-immunoprecipitation of endogenous proteins that the pyrophosphorylation status of IC-2C positively regulates its interaction with p150^Glued^. In other words, in WT MEFs, the association between IC-2C and p150^Glued^ is stronger than that in *ip6k1^−^*^/*−*^ MEFs. Dynactin regulates the binding of dynein to intracellular organelles, such as endosomes and phagosomes, which are then transported along microtubules. Therefore, the IC-2C pyrophosphorylation status affects motor protein docking to vesicles, explaining the impaired dynein-dependent vesicle transport observed in *ip6k^−^*^/*−*^ cells.

The work of Chanduri et al. [9] adds to the small handful of studies that have investigated protein pyrophosphorylation [7–9,11,12]. The pyrophosphorylation of serine residues, while exciting, is a concept surrounded by justified scepticism. The criticism surrounding this post-translational modification is understandable and appropriate, since there is no direct evidence supporting the existence of this post-translational modification *in vivo*. It is relatively simple to verify whether a protein is pyrophosphorylated *in vitro* using radiolabelled 5-[β-^32^P]IP_7_. It is also reasonably simple to perform a ‘back-pyrophosphorylation’ assay. This analysis, however, presupposes that serine pyrophosphorylation exists *in vivo* in the first place; while this seems logical, it might not be the case. Therefore, back-pyrophosphorylation is an indirect assay and prone to uncertainty and criticism. The dissection of another indirect *in vivo* evidence of protein pyrophosphorylation, in-gel mobility shift, resulted in connecting the mobility shift to a different protein post-translational modification, lysine polyphosphorylation, and not to serine pyrophosphorylation [13]. Therefore, it becomes imperative to demonstrate the direct presence of pyrophosphoserine peptides by mass spectrometry. Initially, it would be important to demonstrate the existence of pyrophosphoserine residues in peptides pyrophosphorylated *in vitro*. However, the ultimate proof will come by demonstrating the presence of pyrophosphoserine in proteins extracted from cells, thus pyrophosphorylated *in vivo* by the dynamic inositol pyrophosphate cellular metabolism. These important objectives are getting closer since they are now facilitated by the viability of chemically synthesized inositol pyrophosphates, by their delivery into cells [14,15], and by the development of several newly generated chemical tools that target the synthesis and detection of pyrophosphorylated peptides [16].

## Abbreviations

CK2: casein kinase 2
EEA1: early endosome antigen 1
ERC: endocytic recycling compartment
IC-2C/DYNC1I2: cytoplasmic dynein 1 intermediate chain 2
IP_3_: inositol trisphosphate
5-IP_7_: 5-diphosphoinositol pentakisphosphate
IP6K1: inositol hexakisphosphate kinase 1
MEF: mouse embryonic fibroblast
WT: wild-type.
